# A Single Amino Acid Mutation (R104P) in the E/DRY Motif of GPR40 Impairs Receptor Function

**DOI:** 10.1371/journal.pone.0141303

**Published:** 2015-10-27

**Authors:** Shimeng Guo, Jiandong Zhang, Shuyong Zhang, Jing Li

**Affiliations:** 1 School of life sciences, Shanghai University, Shanghai, China; 2 College of Pharmacy, Nanchang University, Nanchang, China; 3 Chinese Academy of Sciences Key Laboratory of Receptor Research, National Center for Drug Screening, Shanghai Institute of Materia Medica, Chinese Academy of Sciences, Shanghai, China; Rutgers University, UNITED STATES

## Abstract

Type 2 Diabetes Mellitus with insulin resistance, pancreatic β cell dysfunction, and hepatic glucose overproduction is increasing in epidemic proportions worldwide. G protein-coupled receptor 40 (GPR40), a clinically proven anti-diabetic drug target, is mainly expressed in pancreatic β cells and insulin-secreting cell lines. Long chain fatty acids (LCFA) increase intracellular calcium concentration and amplify glucose-stimulated insulin secretion by activating GPR40. Here we report that the arginine 104 (R104) is critical for the normal function of GPR40. Mutation of R104 to Proline (R104P) results in complete loss of the receptor function. Linoleic acid, ligand of GPR40, could not elicit calcium increase and ERK phosphorylation in cells expressing this mutant receptor. Further study indicated the R104P mutation reduces cell surface localization of GPR40 without affecting the expression of the protein. The small portion of GPR40 R104P mutant that is still located on the membrane has no physiological function, and does not internalize in response to linoleic acid stimulation. These data demonstrate that R104 in GPR40 is critically involved in the normal receptor functions. Interestingly, R104P is a registered single-nucleotide polymorphism of GPR40. The relationship of this GPR40 variant and type 2 diabetes warrants further investigation.

## Introduction

Type 2 diabetes, characterized by insulin resistance, pancreatic β cell dysfunction, and impaired incretion responses, is a global health issue [1]. A number of nutrients, hormones and neurotransmitters have been found to influence glucose-stimulated insulin secretion (GSIS) via G protein-coupled receptors (GPCRs), including GPR40, GLP-1R, GPR119, GPR120, CB1/2, and etc [2–7]. GPR40 is mainly expressed in pancreas, especially enriched in islets β-cells [8–9]. Long-chain free fatty acids (LCFA) have been reported to amplify GSIS from pancreatic β-cells by activating GPR40 [9]. GPR40 knockout mice seem to have normal glucose homeostasis, but islets isolated from these mice display a reduced capacity for LCFA to augment GSIS. On the other hand, the transgenic mice overexpressing GPR40 were glucose-intolerant and hypoinsulinemic even when a normal diet was given and progressed rapidly to diabetes [10–11]. These results suggest that GPR40 plays important roles in the maintenance of insulin and glucose homeostasis and in the development of type 2 diabetes.

**Fig 1:**
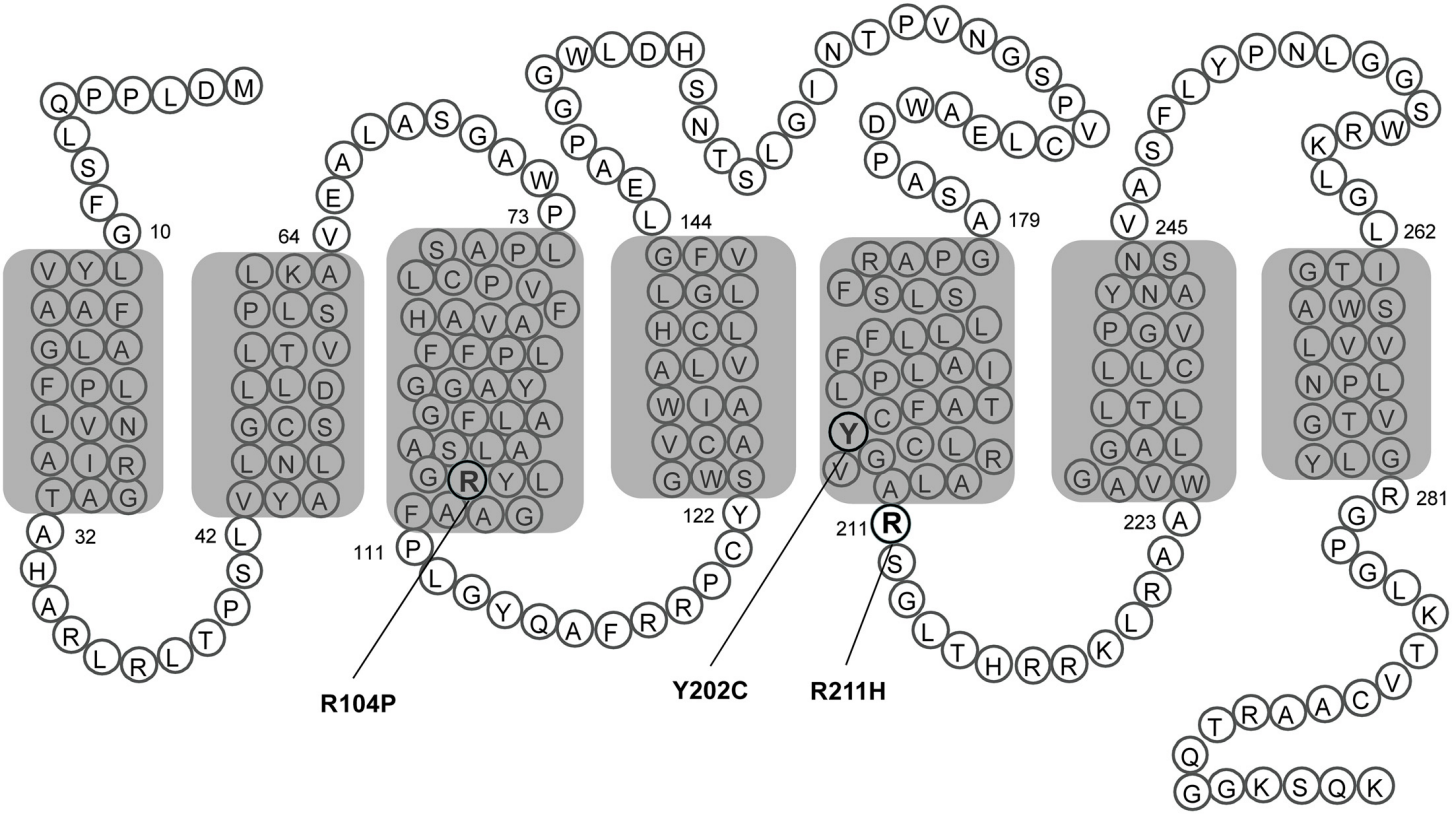
Mutants of GPR40. The point mutations of GPR40 tested in this study are represented as a transmembrane snake plot. The blocked area represents plasma membrane with seven putative transmembrane domains.

GPR40 is an attractive drug target for the treatment of type 2 diabetes. We were interested in searching new GPR40 agonists due to their potential application in therapeutics and signal transduction research. A plasmid encoding human GPR40 was constructed. But unfortunately, upon stimulation with linoleic acid (LA), HEK293 cells transiently transfected with this GPR40 plasmid elicited no calcium response. Sequencing of the GPR40 plasmid revealed triple mutations including R104P, Y202C and R211H (Fig 1). Interestingly, GPR40 R104P and R211H are two SNPs registered in the NCBI database [12], while GPR40 Y202C may be a mutation introduced in the process of PCR. In addition, Arg104 of GPR40 resides at the bottom of the transmembrane helix 3, within the highly conserved E/DRY motif.

Although all GPCRs share a similar topology, sequence analysis does not predict common ligand binding pockets or common binding interface(s) between receptors and G proteins [13]. Therefore the functions of the highly conserved Glu/Asp-Arg-Tyr (E/DRY) motif located at the boundary between transmembrane helix 3 and the second intracellular loop of many GPCRs attracted considerable research attention [14]. This highly conserved motif has been implicated in the regulation of receptor conformational states and/or in the mediation of G protein activation [15–20]. Mutation of the Arg residue in the E/DRY motif affects the function of many GPCRs, such as rhodopsin, adrenergic, histamine, and muscarinic receptors [18–22]. In particular, mutation of the Arg in the E/DRY motif of the vasopressin type II receptor produces a “constitutively desensitized phenotype” with decreased receptor level at the plasma membrane and loss of receptor functions [23]. For other receptors, such as the α2A-adrenergic receptor and the m1 muscarinic receptor, mutation of the Arg decreases agonist binding affinity and impairs signal transduction [19, 21–22].

Although GPR40 R104P is a SNP registered in the NCBI database, its functional impact has never been reported. Here we investigate the effects of the R104P mutation in the E/DRY motif and the other two mutations on the receptor function of GPR40. Our results demonstrate that R104 in GPR40 is critically involved in the normal receptor functions. Mutations in this amino acid may contribute to the loss of function of GPR40 and possibly the development of Type 2 diabetes.

## Materials and Methods

### Materials

Linoleic acid (LA), Docosahexaenoic Acid (DHA), Hoechst 33342 and sulfinpyrazone were purchased from Sigma-Aldrich. AM8596 Antibody against myc-tag (9B11) and horseradish peroxidase (HRP)-conjugated anti-rabbit IgG were purchased from Cell Signaling Technology. Donkey anti-rabbit antibody conjugated to Alexa Fluor 555, and Fluo-4 AM were purchased from Invitrogen.

### Plasmids and mutagenesis

The original mammalian expression vector of hGPR40 carrying R104P, Y202C and R211H mutants was subcloned into a pcDNA3.0 plasmid containing N-terminal Myc-tag. Correction of these mutations was carried out by using the QuickChange Site-Directed Mutagenesis Kit (Stratagene, La Jolla, CA, USA) according to the manufacturer’s instructions. Oligonucleotides for the mutations were as follows: P104R:5’-CCCTGAGTGCAGGCCCCTACCTGGGAGCAGCCTTCCCC-3’; C202Y:5’-GGCCATCACAGCCTTCTGCTGCGTGGGCTGCCTCCGGGCAC-3’; H211R: 5’-GGCTGCCTCCGGGCACTGGCCCACTCCGGCCTGACGCACAGG-3’. The plasmids were sequenced before further investigation.

### Cells culture and transfection

HEK293 cells obtained from American Type Culture Collection were maintained in high glucose DMEM (Life Technologies) supplemented with 10% fetal bovine serum (FBS; Hyclone), 100 mg/L penicillin, and 100 mg/L streptomycin at 37°C in a humidified atmosphere of 5% CO2. HEK293 cells were transfected with plasmids encoding wild type or mutant GPR40 by electroporation. Experiments were carried out 24–36 h after transfection. HEK293 cell lines stably expressing GPR40 were screened with proper antibiotics (400 μg/mL G418 and/or 20 μg/mL blasticidin).

### Calcium mobilization assay

HEK293 cells transfected with wild type or mutant GPR40 were seeded onto a 96-well plate. Then cells were loaded with Fluo-4 AM in HBSS. And LA at various concentrations were dispensed into the well using a FlexStation III microplate reader (Molecular Devices, Sunnyvale, CA, USA), as previously described [4].

### Western blot

HEK293 cells transfected with wild type or mutant GPR40 were serum starved for 24h and then treated with GPR40 ligands for the indicated duration at 37°C. Cells were lysed and sonicated in 1 × SDS buffer. Aliquots of proteins were fractionated by 10% SDS-PAGE and transferred to polyvinylidene difluoride membranes. The membranes were blocked with 5% nonfat milk buffer for 1 h at RT and then incubated overnight at 4°C in buffer containing anti-GAPDH (1:1,500), anti-Myc (1:1,500), anti-ERK (1:2,000), or anti-p-ERK (1:2,000). Immunostaining was visualized using Amersham ECL Plus Western blotting detection reagents and images were taken with a ChemiDoc XRS imaging system (Bio-Rad, Hercules, CA, USA).

### Immunofluorescence microscopy

HEK293 cells transfected with wild type or mutant GPR40 were seeded onto 96-well plates at a density of 3 × 10^4^ per well. The plate was pre-coated with Matrigel (1:100, BD biosciences, San Jose, CA, USA) to facilitate cell attachment. After overnight incubation, stimulation with 100 μM linoleic acid for various time periods, then cells were fixed with 4% formaldehyde in phosphate-buffered saline. For cell surface receptor detection (non-permeabilized condition), cells were incubated with anti-Myc antibody (1:500) and secondary antibody (1:500) conjugated to Alexa Fluor 555 at room temperature for 1 h. For total receptor detection (permeabilized condition), cells permeabilized with 0.3% Triton X-100 after fixed. Finally, the cell nuclei were stained with Hoechst 33342 for 10 min at room temperature. Fluorescent images were obtained with an Olympus IX51 inverted fluorescent microscope. The fluorescent intensity of the cells was measured with a Cellomics ArrayScan 4.0 HCS Reader, which can automatically identify and outline each cell. Experiments were run in triplicate, and about 1,000 cells from each well were analyzed.

### Data analysis

Data were analyzed with GraphPad Prism software (GraphPad). Nonlinear regression analyses were performed to generate dose-response curves and calculate EC_50_ values. Means ± SEM were calculated using this software. Two-tailed Student's t-tests were performed to determine statistically significant differences.

## Results

### GPR40 R104P mutant abolishes LA-stimulated signaling

**Fig 2:**
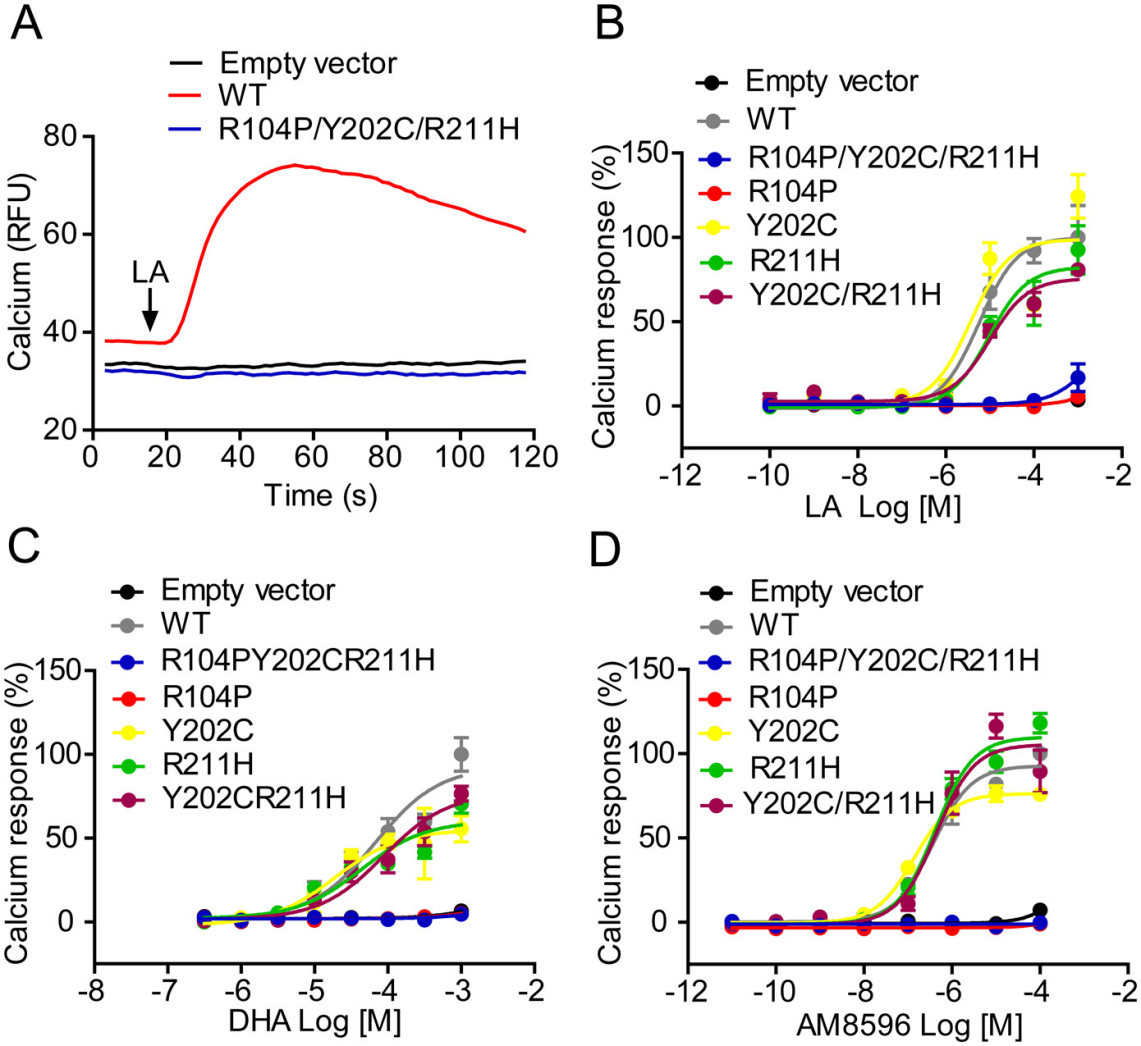
Calcium response in HEK293 cells expressing various GPR40 mutants in response to LA, DHA and AM8596. HEK293 cells were transfected with wild type or mutant GPR40 by electroporation. Cells were then loaded with Fluo-4 AM and intracellular calcium changes after stimulation with different compounds were monitored. (A) Intracellular calcium changes of empty vector, wild-type and R104P/Y202C/R211H mutant hGPR40. (B, C and D) Calcium mobilization in empty vector, wild-type, R104P/Y202C/R211H, R104P, Y202C, R211H and Y202C/R211H in response to LA (B), DHA (C) and AM8596 (D). Data are presented as the means ± SEM (n = 3).

GPR40 has been reported to couple to intracellular Ca2+ signaling via Gαq/11. Activation of GPR40 by fatty acids has been shown to result in an increase in intracellular [Ca^2+^]. To investigate which of the amino acid residues contributed to the loss of function in the original triple mutant of GPR40, plasmids carrying single mutation (R104P, Y202C or R211H) or double mutation (Y202C/R211H) were constructed (Fig 1). Then HEK293 cells were transiently transfected with empty vector, WT or mutant GPR40, and receptor function was assessed with calcium assay. As demonstrated in Fig 2 and Table 1, LA (Fig 2B), DHA (Fig 2C) and AM8596 (Fig 2D) could not elicit calcium response in cells expressing the triple mutant or the R104P mutant, whereas cells expressing Y202C, R211H, or the Y202C/R211H double mutant responded similarly as cells expressing the WT receptor (Fig 2; Table 1).

**Table 1:** EC_50_ values of different ligands on GPR40 mutants measured by calcium mobilization assay.

hGPR40 Mutants	EC_50_ (Mean ± SEM, μM)		
	LA	AM8596	DHA
Empty vector	-	-	-
WT	7.24±2.17	0.43±0.04	60.83±3.63
R104P/Y202C/ R211H	-	-	-
R104P	-	-	-
Y202C	3.92±0.36	0.14±0.01	19.62±0.67
R211H	7.91±0.81	0.39±0.04	44.97±1.21
Y202C/R211H	9.09±0.45	0.43±0.02	79.89±17.6

**Fig 3:**
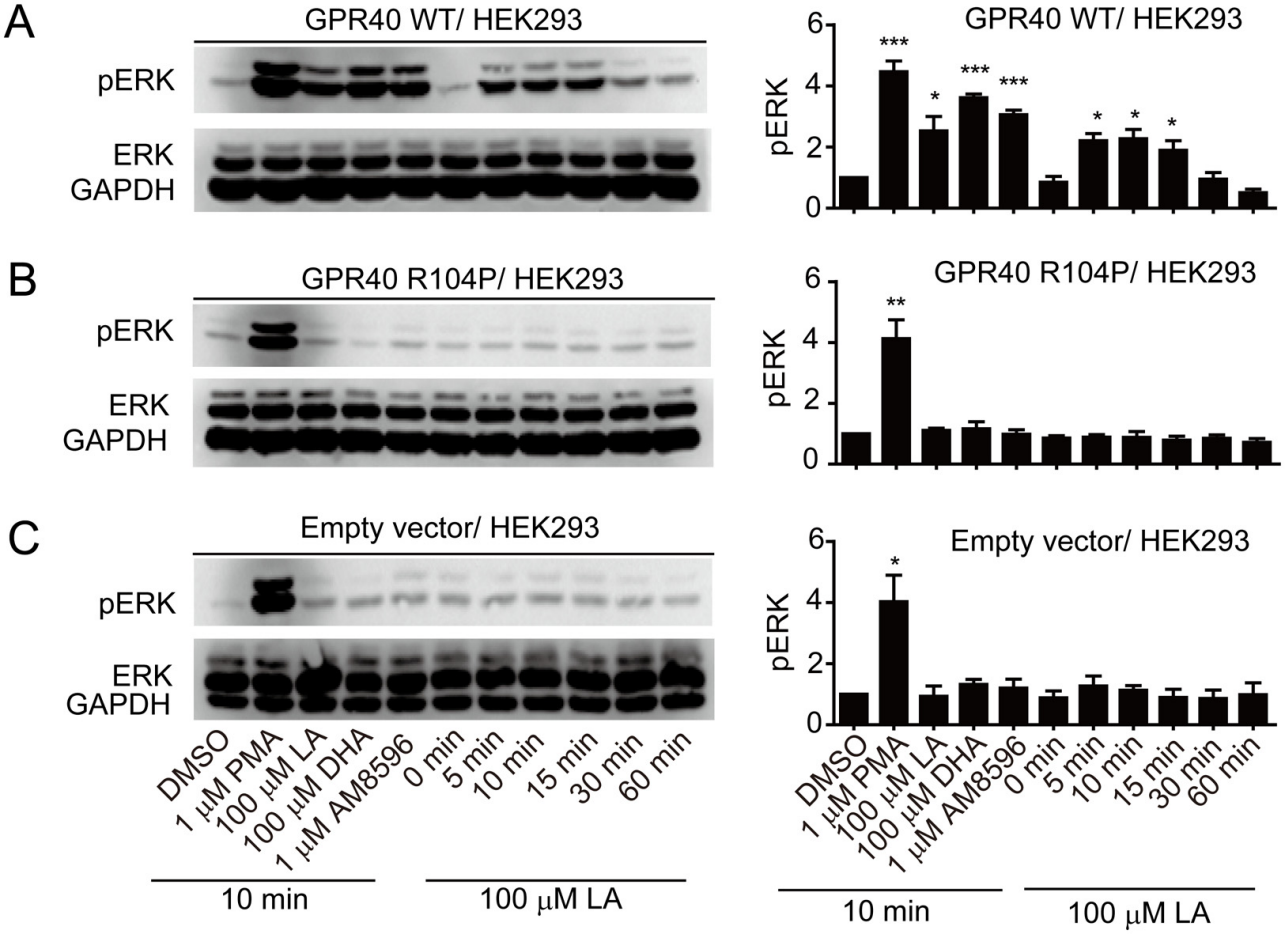
Effect of R104P mutant on GPR40 mediated ERK phosphorylation. HEK293 cells transiently transfected with wild type (A) or R104P mutant (B) or empty vector (C) were stimulated with different ligands at indicated concentration at 10min or 100 μM LA for various durations (0, 5, 10, 15, 30 and 60 min) at 37°C, and ERK1/2 phosphorylation was detected with Western blot. ERK phosphorylation levels were normalized to the GAPDH level in the same sample. Data are means ± SEM (n = 3). *P < 0.05, **P < 0.01 and ***P < 0.001, versus vehicle control.

Many GPCRs also couple to the extracellular signal regulated protein kinases (ERK) pathway. To test whether R104P mutation affects ERK activation, HEK293 cells were transiently transfected with plasmids encoding empty vector, WT or R104P mutant. Then these cells were stimulated with different ligands at indicated concentration at 10min or 100 μM LA for various durations (0, 5, 10, 15, 30 or 60 min). Different GPR40 ligands dramatically induced phosphorylation of ERK1/2 in HEK293 cells expressing the WT receptor, and LA induced phosphorylation of ERK1/2 in a time-dependent manner the signal peaked at 10 min of stimulation (Fig 3A). In contrast, GPR40 ligands had no effect in HEK293 cells transfected with R104P mutant or empty vector (Fig 3B and 3C). These results suggest that R104P mutation abolishes GPR40-mediated ERK activation.

### R104P mutation reduces cell surface but not total GPR40 protein level

**Fig 4:**
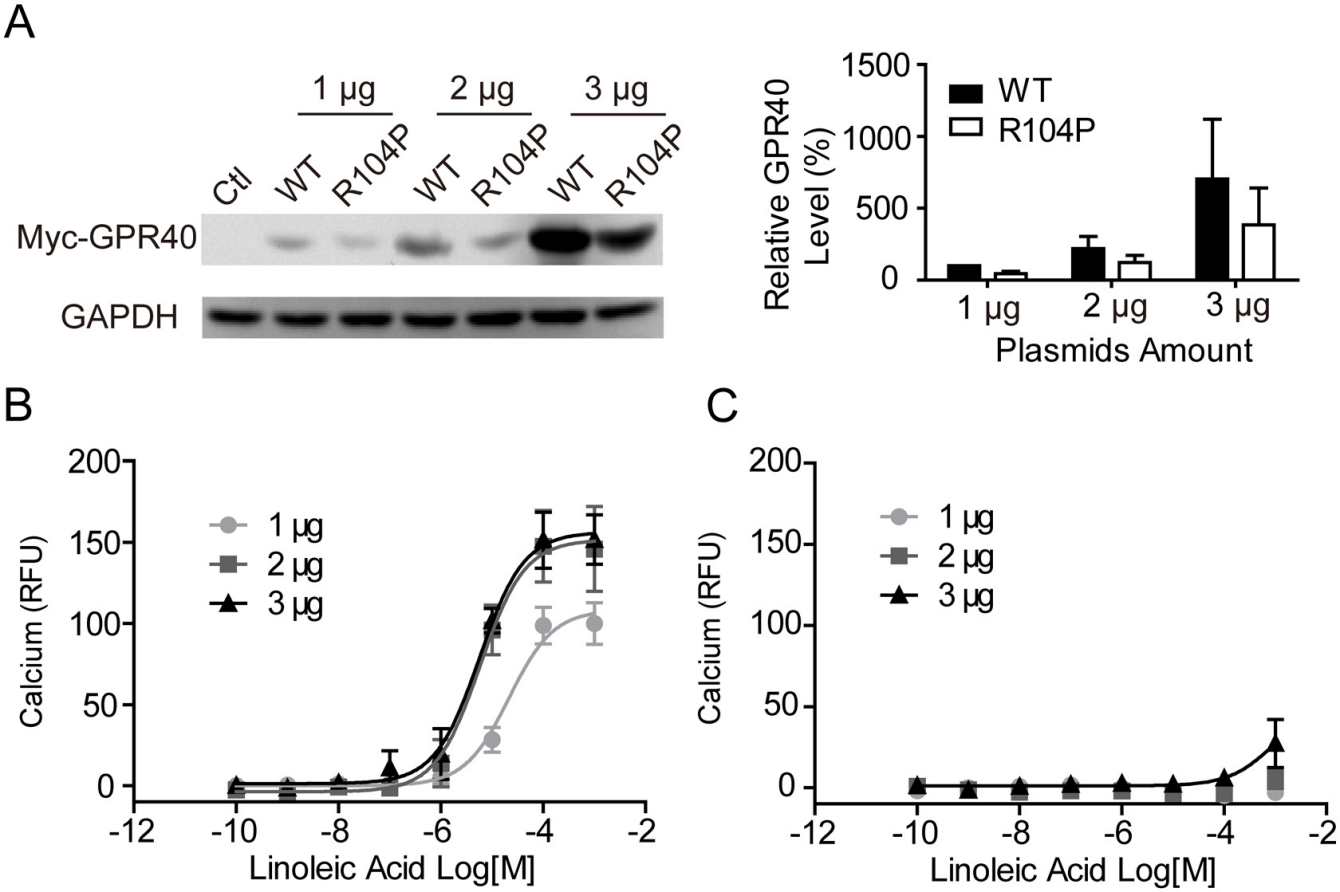
Effect of expression of GPR40 in HEK 293 cells on calcium response. (A) HEK293 cells were transiently transfected with different amount of plasmids (wild type or R104P mutant), and GPR40 was detected with Western blot. (B and C) HEK-293 cells were transfected with wild type (B) or R104P mutant (C), then cells were loaded with Fluo-4 AM and intracellular calcium changes after stimulation with linoleic acid were monitored. GAPDH, glyceraldehyde-3-phosphate dehydrogenase.

To ensure that the phenomena we observed was not due to the expression difference between the WT and R104P mutant, HEK293 cells were transiently transfected with various amount plasmids encoding myc-tagged WT or R104P GPR40. Western blot analysis of the whole cell lysates revealed that protein level of R104P mutant was only slightly reduced comparing to the WT receptor (Fig 4A). Meanwhile, HEK293 cells transfected with different amount of GPR40 plasmids were tested in calcium assay. For WT GPR40, reduced amount of plasmids did lead to reduced protein level (Fig 4A) and reduced calcium response after LA stimulation (Fig 4B). But for the R104P mutant, even the highest amount of plasmids (3 μg) resulted in no calcium response (Fig 4C), although the protein expression level in cells transfected with 3 μg R104P was much higher than those transfected with 2 μg WT plasmid (Fig 4A). These results suggest that R104P mutation do not affect overall GPR40 protein level.

**Fig 5:**
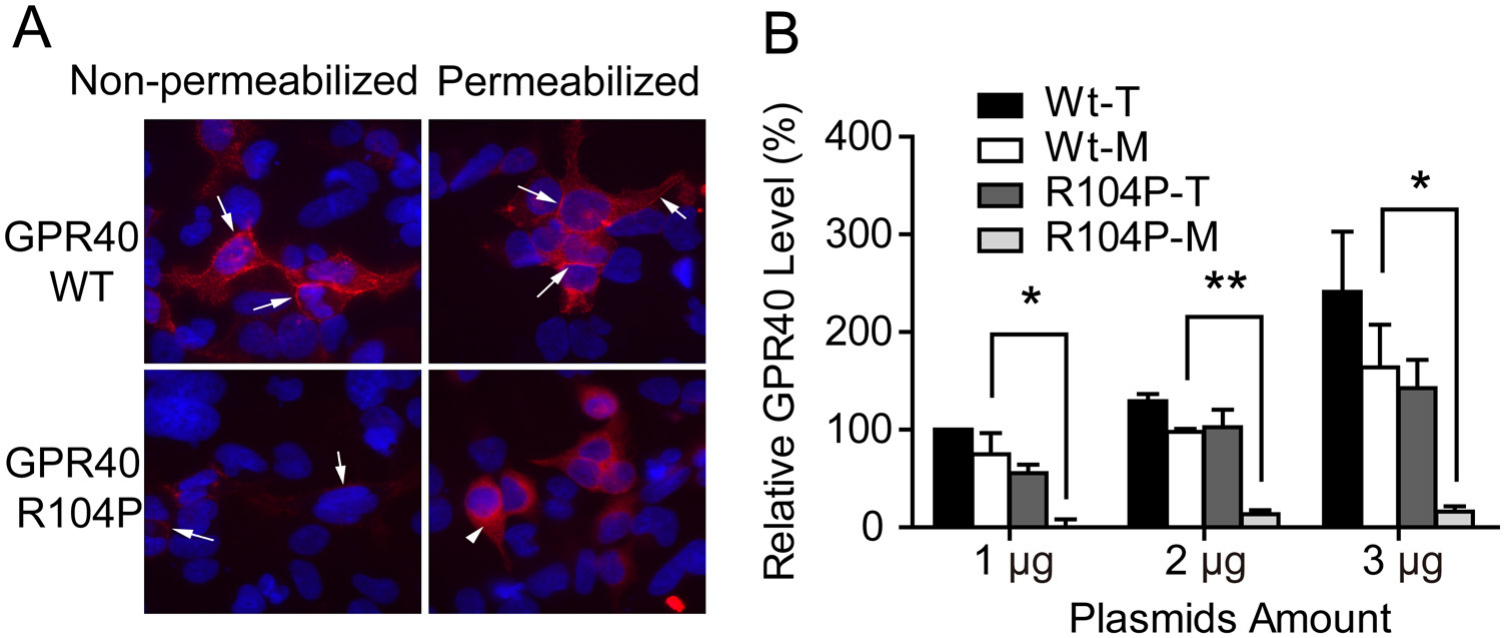
Effect of R104P mutant on cell surface localization of GPR40. (A) Translocation of GPR40 was studied with HEK293 cells transiently transfected with wild type or R104P mutant, and Cells were stained with non-permeabilized and permeabilized condition. GPR40 membranes were stained with myc-tagged GPR40 (red, arrows). Cell nuclei were stained with Hoechst 33342 (blue), 2 μg of DNA transfected. (B) Quantitative analysis of wild type and R104P mutant expression with plasmid of different amount as measured by membranes (M) and total protein (T), (shown in panel A). Data are means ± SEM (n = 3). *P < 0.05 and **P < 0.01, WT membranes versus R104P membranes. Scale bar, 10 μm.

Since GPCR must be located in the plasma membrane to have proper functions, we wondered whether R104P mutation affect GPR40 membrane localization. The cell surface or total GPR40 levels were detected using immunofluorescent staining in non-permeabilized or permeabilized cells. The majority of the WT GPR40 could be detected in the cell surface of the non-permeabilized cells, while most of the R104P mutant receptor could only be detected in permeabilized cells (Fig 5A). Statistical analysis of fluorescent intensity revealed ~70% of the WT GPR40 located on the cell membrane, while only ~10% of the R104P mutant located on the cell membrane (Fig 5B). These results strongly suggest that the R104P mutation does not affect overall GPR40 protein level, but reduces membrane localization of this receptor.

### GPR40 R104P mutant do not internalize upon stimulation

**Fig 6:**
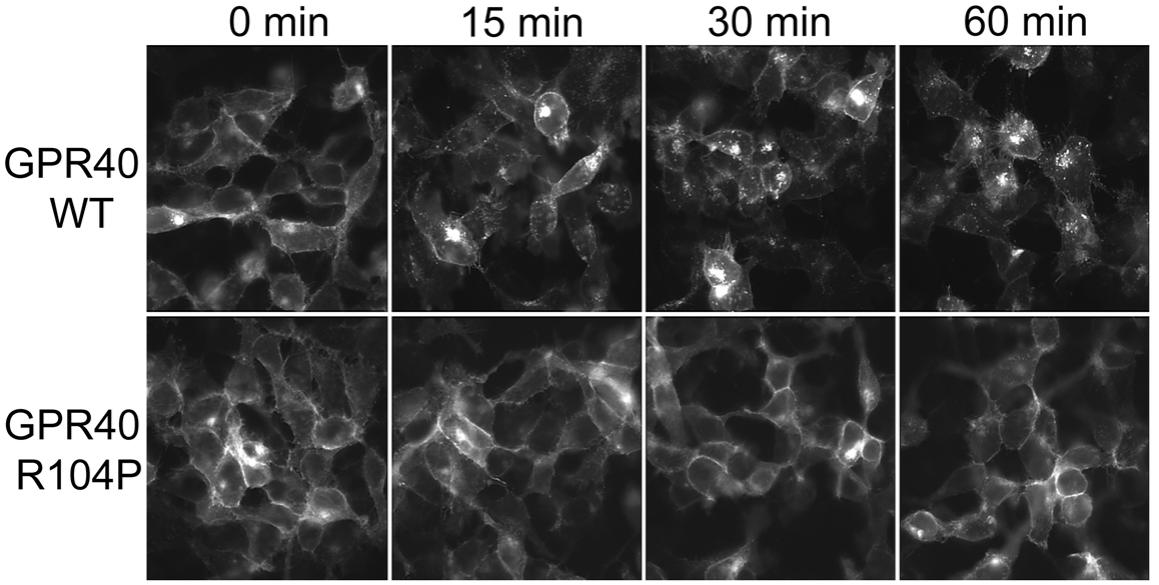
Linoleic acid induces internalization of GPR40. HEK293 cell lines stably expressing with myc-tagged wild type or R104P mutant, were incubated with 100 μM Linoleic acid for 0–60 min at 37°C and stained with permeabilized condition. And the cell-surface level of GPR40 was determined by measuring surface myc immunoreactivity with Immunofluorescence microscopy.

Receptor internalization is another common phenomenon after agonist stimulation [24]. Although the R104P mutation greatly reduces GPR40 level in the plasma membrane, there’s still a small portion of the mutant receptors remained on the cell surface. To study these correctly located mutant receptors, we selected a HEK293 cell line in which the cell surface R104P GPR40 could be clearly visualized by immunostaining (Fig 6). However, these mutant receptors located on the cell surface did not respond to the stimulation of LA (100 μM), while WT GPR40 displayed a clear time-dependent internalization upon LA stimulation (Fig 6). Therefore, these results show that the R104P mutation affects GPR40 internalization.

## Discussion

GPR40, a member of G protein-coupled receptors, is a 300-amino acid protein containing the characteristic 7 transmembrane domains. In GPR40, R104 is located at the boundary of transmembrane helix 3 and intracellular loop 2. P202 is located at the boundary of transmembrane helix 5 and intracellular loop 3. And the R211 is located between transmembrane helices 5 and 6 [25]. Medium- to long-chain saturated and unsaturated FFAs were able to induce an elevation of intracellular calcium concentration in human GPR40-expressing HEK293 cells [8–9, 26]. GPR40 is mainly expressed in the pancreas, especially enriched in islets β-cells [8–9].

Abecasis et al identified twenty-nine missense variants of human GPR40 [27]. The most common human SNP has been identified in the coding region of GPR40 is R211H [28]. It is reported that R211H polymorphism might contribute to the variation of insulin secretory capacity in healthy Japanese men [29]. However, later studies reported that R211H has no correlation with metabolic abnormalities [26]. Smith et al revealed that WT and R211H variant displayed equal efficacy in binding assay [30]. In calcium measurements, GPR40 agonists produced equivalent responses at WT and R211H variant, consistent with our results. Other SNPs, such as D175N and G180S, have also been reported to reduce the efficacy of GPR40 in vitro [31–32]. However, other study found that the pharmacology of these variants is not different from the wild type receptor [30].

Our results have shown that the R104P mutation in the E/DRY motif of GPR40 strongly impairs agonist-induced, Gαq/11-dependent, intracellular calcium mobilization, whereas Y202C and R211H mutations have no significant impact on calcium response. This loss-of-function phenotype seems to be caused by reduced receptor presence in the plasma membrane, indicating that R104 in the E/DRY motif is a key residue for proper localization of GPR40. The E/DRY motif is directly involved in regulating receptor conformation and G protein coupling/recognition. Mutations of the amino acids within this motif have been demonstrated to impair agonist-induced responses in many GPCRs [33]. Our findings suggest that this highly conserved motif also play a critical role in regulating GPR40 functions.

In summary, we identified that the R104 in the E/DRY motif of GPR40 play critical role in receptor function. Mutation in this residue results in a loss of agonist-induced functions, including calcium mobilization, ERK activation and receptor internalization. Since R104P is a reported SNP of GPR40, variation in this amino acid residue may contribute to the loss of function of GPR40. However, the relationship of such a GPR40 variant to obesity and type 2 diabetes remains to be established. Future studies in vitro and in vivo may further clarify the role of the polymorphism in mouse and human β-cell function.
